# Efficacy of Indocyanine Green Fluorescence Angiography in Preventing Anastomotic Leakage After McKeown Minimally Invasive Esophagectomy

**DOI:** 10.3389/fonc.2020.619822

**Published:** 2021-01-08

**Authors:** Rao-Jun Luo, Zi-Yi Zhu, Zheng-Fu He, Yong Xu, Yun-Zheng Wang, Ping Chen

**Affiliations:** ^1^ Department of Thoracic Surgery, Sir Run Run Shaw Hospital, School of Medicine, Zhejiang University, Hangzhou, China; ^2^ Zhejiang Provincial Key Laboratory of Laparoscopic Technology, Sir Run Run Shaw Hospital, School of Medicine, Zhejiang University, Hangzhou, China

**Keywords:** gastric conduit, McKeown, minimally invasive esophagectomy, indocyanine green fluorescence angiography, anastomotic leakage

## Abstract

**Background:**

Indocyanine green (ICG) fluorescence angiography (FA) was introduced to provide real-time intraoperative evaluation of the vascular perfusion of the gastric conduit during esophagectomy. However, its efficacy has not yet been proven. The aim of this study was to assess the usefulness of ICG-FA in the reduction of the rates of anastomotic leakage (AL) in McKeown minimally invasive esophagectomy (MIE).

**Methods:**

From June 2017 to December 2019, patients aged between 18 and 80 years with esophageal carcinoma were enrolled in the study and each patient underwent McKeown MIE. Patients were divided into two groups, those with or without ICG-FA. The patient demographics and perioperative outcomes were comparable between the two groups. The primary outcome was the rate of AL.

**Results:**

A total of 192 patients were included: 86 in the ICG-FA group and 106 in the non-ICG-FA group. Overall, 12 patients (6.3%) had AL; the rate of AL was 10.4% in the non-ICG-FA group, which was significantly higher than the 1.2% in the ICG-FA group.

**Conclusions:**

ICG-FA has the potential to reduce the rate of AL in McKeown MIE.

## Introduction

Esophageal cancer is the eighth most common cancer and the sixth leading cause of cancer-related death worldwide (1, 2). The incidence of esophageal cancer in China ranks fifth in terms of malignant tumors and the mortality rate ranks fourth (3, 4). Esophagectomy is still the mainstream multidisciplinary treatment for esophageal cancer in combination with chemoradiotherapy (4). Esophagectomy is an invasive and complicated procedure, and the frequency of postoperative complications is as high as 59% (5). Anastomotic leakage (AL) after esophagectomy is one of the most dreaded postoperative complications, and is responsible for higher postoperative morbidity and mortality rates, prolonged hospital stays, and the need for further surgical procedures (6, 7).

Several risk factors are involved in the development of AL; some are unchangeable, such as age, body mass index (BMI), comorbidities, and advanced tumor stage, while others, such as anastomotic tension, location of anastomosis, surgical technique, and postoperative factors, are modifiable, and many efforts have been made to decrease AL in the last decades (8–10). However, vascular perfusion to the anastomotic regions is considered to be the main driving force of AL (7, 10). In the construction of gastric conduits, it is difficult for the gastroepiploic arcade to reach the distal end of the conduit. The blood supply to the rest of the gastric tube is mainly provided by submucosal vessels. Evaluation of vascular perfusion in the area undergoing anastomosis depends largely on the objective identification of the gastric serosa by surgeons. Therefore, correctly identifying the anastomosis perfusion and selecting an appropriate anastomotic site are important to reduce AL (11).

As a means of evaluating blood flow, indocyanine green (ICG) fluorescence angiography (FA) has recently been introduced to provide real-time assessment of the anastomosis area during esophagectomy (11–13). ICG is a hydrophilic tricarbonyl cyanine dye that quickly binds to plasma proteins after intravenous injection. When the ICG binding protein is excited by a light source at 750 to 810 nm, it emits peak fluorescence around 840 nm and allows for the detection of signals as deep as 10 mm within the tissue. Therefore, ICG can be used as both a lymphatic and vascular contrast agent (14).

Although some studies have reported that ICG-FA is a potentially promising method to assess gastric vascular perfusion, these studies have limitations such as a small population study, the use of various surgical methods, or lack of a control group (10–17). Therefore, it is still unclear whether ICG-FA can decrease the rate of AL during esophagectomy. As a retrospective cohort trial, the aim of our study was to investigate the real-time perfusion of the conduit using ICG-FA during esophageal procedures. In addition, we sought to assess the efficacy of ICG-FA in the reduction of AL rates in McKeown minimally invasive esophagectomy (MIE).

## Materials and Methods

### Patients

This retrospective cohort study included 192 consecutive patients with esophageal carcinoma who underwent McKeown MIE with cervical anastomosis between January 2017 and December 2019 at our hospital. Patients aged between 18 and 80 years with histologically proven primary esophageal carcinoma that was considered to be resectable were qualified. All patients underwent routine examinations, including routine blood tests, blood biochemical examinations, and electrocardiograms. Computed tomography of the chest and abdominal (CT), positron emission tomography (PET-CT), and B-ultrasound were implemented to rule out cervical lymph node metastasis. Patients with a previous history of gastrointestinal or lung cancer, severe comorbidities, other organ metastasis, combined other organ resection, or allergic hypersensitivity to ICG or iodine were excluded. The patients’ treatment strategies were formulated by a multidisciplinary expert team, and all patients’ operations were carried out by the same surgical team. The research protocol for this clinical study was approved by the local ethics committee. Evaluation of gastric vascular perfusion at the anastomosis sites using ICG fluorescence angiography initiated in June 2018 when an advanced laparoscopic system equipped with near-infrared camera was introduced. Since then, the ICG-FA system has been used in all patients, except for patients with allergic hypersensitivity to ICG or when the ICG-FA system was occupied by other surgical groups.

### Analysis

The patients were divided into two groups: Patients who did not undergo ICG-FA were classified as the non-ICG-FA group, and patients who received ICG-FA were classified as the ICG-FA group. The collected individual and tumor-related characteristics included age, sex, BMI, comorbidities, history of smoking, history of drinking, American Society of Anesthesiologists (ASA), histopathology details, and tumor location. Information on preoperative therapies, including neoadjuvant radiotherapy (NRC), neoadjuvant chemotherapy (NAC), and endoscopic submucosal dissection (ESD), was also collected. Perioperative outcomes and complications were assessed. The primary endpoint was the rate of AL within 30 days after the initial surgery, or the rate of AL before the patient recovered from discharge. AL was defined as extravasation of contrast on postoperative CT image, presence of empyema on chest CT, or elevated drain amylase level with clinical evidence of leak (11). Postoperative electronic gastroscopy was arranged to inspect the situation around the anastomosis in suspected individuals. The secondary outcomes were operative time, blood loss, time until restoration of fluid diet, length of hospital stay, postoperative complications and oncological clearance.

### Surgical Techniques

In our institution, we performed McKeown MIE with cervical anastomosis for esophageal cancer. The details of these procedures have been described previously (18). Briefly, disconnection of the azygous vein, incision of the mediastinal pleura, and dissection of the paraoesophageal and mediastinal lymph nodes was completed. The thoracic esophagus was mobilized from the azygous arch to the diaphragm hiatus while protecting the thoracic duct. The patients were then placed in a lithotomy position, and the gastrocolic ligament was separated along the greater curvature with protection of the gastroepiploic vascular arch (19). The gastric conduit was closed with an Endo-GIA stapler (ECR60, Echelon, USA) through a 5-cm mini-incision in the upper abdomen and the appropriate width of the gastric conduit was 3–4 cm based on our experience (18). Seromuscular layer sewing was routinely preferred. The cervical esophagus was mobilized after making an oblique incision on the left neck, and the gastric conduit was pulled up through the esophageal bed to the left neck incision for gastroesophageal end-to-side anastomosis (SDH 25A or SDH 21A, Ethicon Endo-Surgery, USA).

### ICG Fluorescence Angiograph

**Figure 1:**
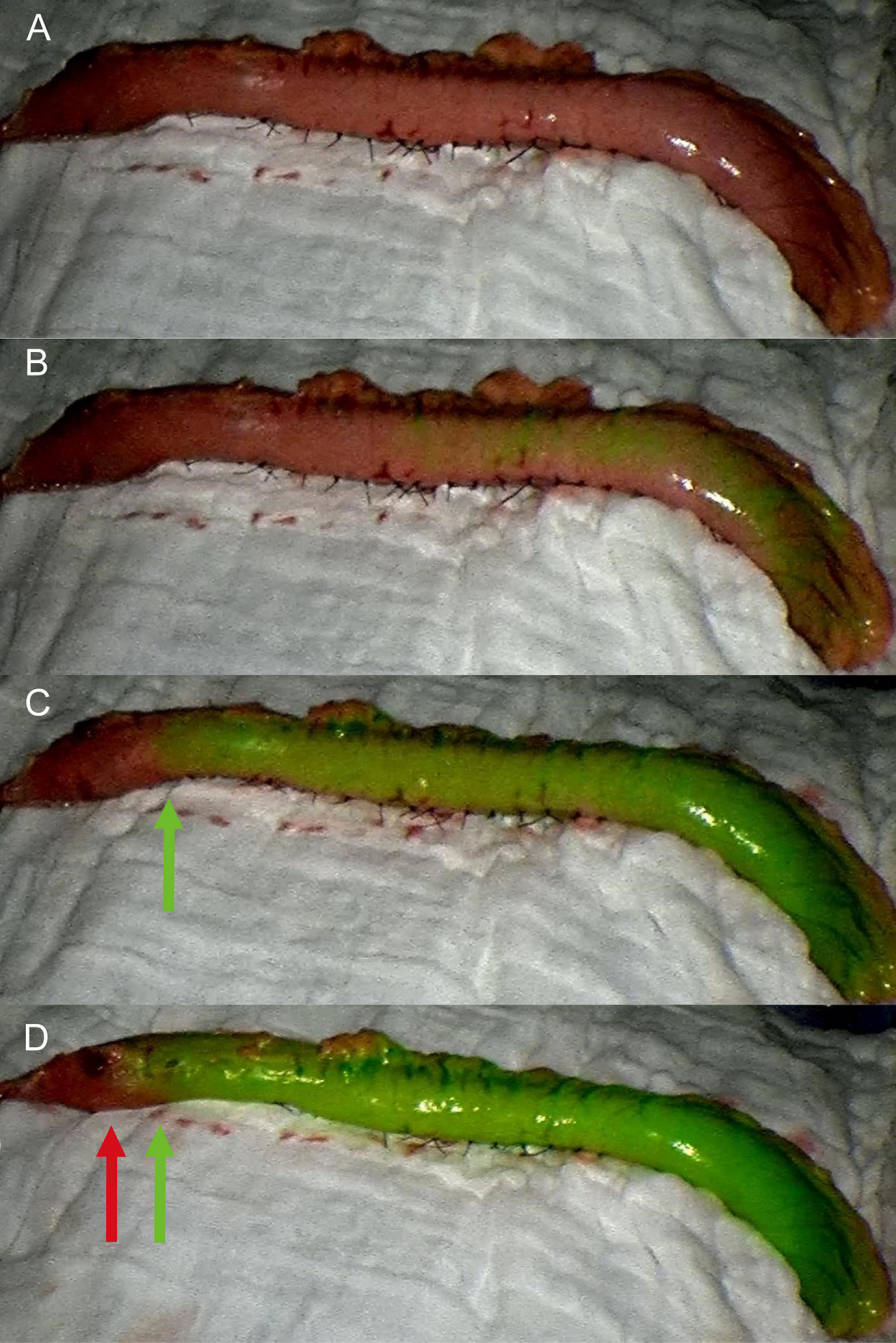
Fluorescence evaluation during McKeown MIE. **(A)** The gastric conduit was pulled up and placed on the anterior chest wall. **(B)** Fluorescence was observed at the root of the conduit approximately 20 s after the injection. **(C)** The good perfusion zone was visualized within 60 s, and anastomosis was performed within the planned transection line (green arrow). **(D)** No vascular perfusion or perfusion times > 60 s were considered to indicate the poor perfusion zone (red arrow).

The gastric conduit was created outside the abdominal cavity and placed on the anterior chest wall (**Figure 1A**). Surgeons checked the conditions of the gastric conduit, such as the color of the gastric serosa, arterial pulsation of the gastroepiploic artery, and the connection between the right and left gastroepiploic vessels (16). Subsequently, 0.5 mg/kg or a maximum of 25 mg of ICG dye (Diagnostic Green, Dandong Yichuang Pharmaceutical, Liaoning, China) was diluted in 10 cc of sterile water, and was injected as a bolus through a central venous catheter by assistant. A near-infrared camera system (Novadaq Technologies Inc. Richmond, Canada) was used for detecting the ICG fluorescence. The surgeon hold the camera 20 cm away from the gastric conduit and the evaluation with fluorescence was performed in real-time. Approximately 20 s after injection, blood flow was confirmed by ICG-FA as it passed through the right gastroepiploic vessels (**Figure 1B**). If vascular perfusion *via* ICG-FA was well visualized within 60 s, it was defined as a good perfusion zone (**Figure 1C**), while no vascular perfusion or perfusion times > 60 s were considered to indicate a poor perfusion zone (**Figure 1D**). The anastomotic perfusion result using ICG-FA was subjectively evaluated by the surgical team and the anastomosis was performed within the good perfusion zone. After the anastomosis, the poorly perfused zone of conduit was removed. The aforementioned area referred to the area where the perfusion time exceeds 60 s, such as the area at the far end of the green arrow in the figure (**Figure 1D**). We created the anastomosis in accordance with the 60 s rule.

### Statistical Analysis

Descriptive statistics were used to depict the demographic parameters of the patients. Measurement data are expressed as mean ± standard deviation. The independent sample t-test was used for comparison between groups, while Mann–Whitney U and Pearson’s χ2 tests were performed to test the difference between the two groups. Statistical analysis was completed using SPSS^®^ version 22 (IBM Corp. in Armonk, NY, USA), with p < 0.05 indicating a statistically significant difference.

## Results

### Demographic Parameters

A total of 192 patients were included in this study. ICG-FA was performed in 86 cases (ICG-FA group) and was not performed in 106 cases (non-ICG-FA group). The patient and tumor characteristics of the entire cohort are shown in **Table 1**. The baseline characteristics were similar between the two groups, and there were no significant differences in age, sex, BMI, history of smoking, history of drinking, ASA, comorbidities, neoadjuvant chemotherapy, neoadjuvant radiotherapy, history of ESD, or tumor location. In both groups, squamous cell carcinoma was the most common pathological type. There were two patients with esophageal adenocarcinoma (2.3%) in the ICG-FA group and five patients with other pathological types (4.7%) in the non-ICG-FA group (p = 0.038), including two with sarcomatoid carcinomas, one with adenosquamous carcinoma, one with malignant melanoma, and one with neuroendocrine carcinoma.

**Table 1:** Demographic parameters and tumor characteristics of the patients.

	ICG-FA(n = 86)	Non-ICG-FA(n = 106)	*P*-value
Age (years)*	65.86 ± 6.54	64.94 ± 6.91	0.350
Sex, n (%) Male Female	74(86.0) 12(14.0)	97(91.5) 9(8.5)	0.252
BMI (kg/m^2^)*	22.62 ± 2.97	22.05 ± 3.36	0.220
History of smoking, n (%) No Yes	49(57.0) 37(43.0)	54(50.9) 52(49.1)	0.404
History of drinking, n (%) No Yes	46(53.5) 40(46.5)	55(51.9) 51(48.1)	0.825
ASA, n (%) II III	79(91.9) 7(8.1)	102(96.2) 4(3.8)	0.224
Comorbidity, n (%) Hypertension Diabetes mellitus Cardiovascular disease Obstructive lung disease Cerebrovascular disease	30(34.9) 4(4.7) 5(5.8) 1(1.2) 3(3.5)	31(29.2) 4(3.8) 4(3.8) 5(4.7) 1(0.9)	0.438 0.762 0.517 0.227 0.327
Neoadjuvant chemotherapy, n (%)	13(15.1)	12(11.3)	0.519
Neoadjuvant radiotherapy, n (%)	1(1.2)	0(0)	0.448
Endoscopic submucosal dissection, n (%)	3(3.5)	0(0)	0.088
Histological type, n (%) Squamous cell carcinoma Adenocarcinoma Others	84(97.7) 2(2.3) 0(0)	101(95.3) 0(0) 5(4.7)	0.038^a^
Tumor location, n (%) Upper Middle Lower	2(2.3) 35(40.7) 49(57)	8(7.5) 50(47.2) 48(45.3)	0.121

ASA, American Society of Anesthesiologists; BMI, Body mass index.

*Values are expressed as mean ± SD.

^a^statistically significant.

### Operative Outcomes and Pathological Characteristics

The operative outcomes and pathological characteristics are summarized in **Table 2**. The operative time was 249.77 ± 80.80 min for the ICG-FA group and 242.58 ± 57.92 min for the non-ICG-FA group. The intraoperative blood loss was 105.47 ± 57.12 ml for the ICG-FA group and 109.91 ± 76.13 ml for the non-ICG-FA group. There were no significant differences between the two groups in terms of postoperative hospital stay, fluid diet time, number of lymph node dissections, TNM stage, and vascular invasion. In addition, there were 20 patients with perineural invasion in the ICG-FA group, which was higher than that in the non-ICG-FA group (20 vs. 10, p = 0.010).

**Table 2:** Operative and pathological outcomes of the ICG-FA group compared to the non-ICG-FA group.

	ICG-FA(n = 86)	Non-ICG-FA(n = 106)	*P*-value
Operation time (min)*	249.77 ± 80.80	242.58 ± 57.92	0.475
Intraoperative blood loss (ml)*	105.47 ± 57.12	109.91 ± 76.13	0.655
Restore fluid diet time (d)*	9.90 ± 3.64	12.30 ± 12.06	0.076
Postoperative hospital stay (d)*	13.85 ± 10.72	16.74 ± 17.24	0.177
Lymph node dissection (n)*	28.92 ± 13.86	30.07 ± 14.02	0.571
Pathological tumor category, n (%) T1 T2 T3 T4	20(23.3) 20(23.3) 46(53.5) 0(0)	33(31.1) 15(14.2) 57(53.8) 1(0.9)	0.257
Pathological node category, n (%) N0 N1 N2 N3	46(53.5) 25(29.1) 13(15.1) 2(2.3)	52(49.1) 30(28.3) 19(17.9) 5(4.7)	0.762
TNM stage, n (%) Stage I Stage II Stage III Stage IV	28(32.6) 22(25.6) 34(39.5) 2(2.3)	32(30.2) 26(24.5) 42(39.6) 6(5.7)	0.712
Vascular invasion, n (%) No Yes	74(86.0) 12(14.0)	95(89.6) 11(10.4)	0.506
Perineural invasion, n (%) No Yes	66(76.7) 20(23.3)	96(90.6) 10(9.4)	0.010^a^

*Values are expressed as mean ± SD.

^a^Statistically significant difference.

### Postoperative Complications

Details about the postoperative complications are presented in **Table 3**. One patient suffered from AL in the ICG-FA group and died of cancer progression and cachexia 105 days after surgery. The rate of AL was 1.2% in the ICG-FA group, which was significantly lower than the rate of 10.4% in the non-ICG-FA group (p = 0.009). According to the Clavien–Dindo classification, all of the patients in the non-ICG-FA group were classified as grade group IIIa. In addition to regular treatments, including fasting, total parenteral nutrition and anti-infection, they recovered after receiving endoscopic tube placement, irrigation or other non-surgical treatments. None of them underwent secondary surgery due to AL. The rate of vocal cord paralysis was not significantly different between the groups (1.2% vs. 1.9%, p > 0.05); all patients recovered with conservative treatment within 6 months after surgery. In the non-ICG-FA group, two patients underwent secondary surgery: One underwent a debridement suture due to a severe abdominal incision infection, and one case with chyle leak was recovered after secondary operation for thoracic duct ligation. There were no significant differences in the incidence of complications between the two groups, with the exception of the incidence of AL.

**Table 3:** Postoperative complications of the ICG-FA group compared to those of the non-ICG-FA^a^ group.

	ICG-FA (n = 86)		Non-ICG-FA (n = 106)		*P*-value
Anastomosis leakage	1(1.2)		11(10.4)		0.009^b^
Vocal cord paralysis	1(1.2)		2(1.9)		0.687
Wound infection	3(3.5)		3(2.8)		0.794
Pleural effusion	11(12.8)		5(4.7)		0.064
Pneumonia	2(2.3)		5(4.7)		0.463
Chylothorax	1(1.2)		2(1.9)		0.687
Arrhythmia	0(0)		1(0.9)		0.366
Pneumothorax	1(1.2)		4(3.8)		0.259
ICU stay	1(1.2)		3(2.8)		0.421
Secondary surgery	0(0)		2(1.9)		0.200
Death	1(1.2)		0(0)		0.448

^a^Data are presented as n (%).

^b^Statistically significant difference.

## Discussion

In this retrospective study, we found that ICG-FA is an effective strategy to prevent the occurrence of AL following McKeown MIE. Our results indicate that routine use of this method during operation could potentially improve the outcomes of McKeown MIE, and is a promising technology to evaluate the blood supply of the gastric conduit.

Previous studies have pointed out that many factors are likely to be associated with AL, including patient-related characteristics, intraoperative factors, and postoperative factors (8–10, 20). However, some factors, such as anastomotic tension, location of the anastomosis, and the surgical technique are modifiable. Blood perfusion remains the most important factor for performing anastomosis safely during esophagectomy, and it is essential to identify sufficient perfusion of the conduit if an appropriate method for assessing perfusion is available. Traditionally, perfusion of the conduit has been evaluated subjectively by surgeons based on several clinical signs, including the color of the conduit, palpable pulsation of the vessels, peristaltic movement, and active bleeding from marginal arteries (21). However, the above signs and characteristics might be inaccurate, as the assessment relies on the referral of the surgeon and tends to be inconsistent. Thus, an intraoperative tissue blood perfusion evaluation system needs to be established, in which the surgeon can receive objective and reliable information to select an appropriate anastomosis site.

Several previous techniques for assessing blood perfusion of the conduit include color Doppler ultrasound, laser Doppler flow meters, angiography, and oxygen spectroscopy. However, these technologies have not been widely accepted because of the cost of equipment, technical difficulties, and lack of reproducibility (11, 22). Recently, a near-infrared system named ICG-FA was used to perform intraoperative angiography and easily and objectively identify conduits with sufficient perfusion. Some papers have reported that ICG-FA might decrease the incidence of AL by assessing real-time blood flow during esophagectomy. However, these studies had certain limitations such as a small population under study and lack of a control group (10–17). Furthermore, due to the lack of standardization of the surgeon, technique, or conduit for evaluating ICG-FA, the consistency of the conclusions cannot be guaranteed. Thus, we conducted this research to overcome the limitations of previous studies. All patients underwent McKeown MIE with cervical anastomosis using a circular stapler by the same surgical team.

Takeuchi reported an AL rate of 13.3% in 5,354 patients undergoing radical esophageal cancer surgery (7). Karampinis (12) made all efforts to perform anastomosis in the zone of optimal perfusion and found a significant improvement in AL (18.2% vs. 3.0%) compared to the internal controls of the previous 55 patients who underwent esophagectomy. In our study, all patients in ICG-FA group were successful in fluorescence angiography. For 32 patients of them, we found that the whole conduit was well visualized within 60 s, which means that these conduits did not have poorly perfused areas. As for the remaining 54 patients, there is a poorly perfused area of various lengths at the distal end of the conduit according to the 60 s rule. In the ICG-FA group, anastomosis was operated in the site of conduit in which green fluorescence was observed within 60 s. The poorly perfused zone of conduit was removed after the anastomosis. The rate of AL for esophageal cancer resection was 10.4% in the non-ICG-FA group, and was significantly higher than the 1.2% in the ICG-FA group, suggesting that a reduction in the rate of AL of approximately 9% could be achieved by the use of ICG-FA. This evidence strongly demonstrates that sufficient blood perfusion of the gastric conduit is critical for thorough tissue healing at the anastomosis site.

Static blood perfusion of the gastric conduit by ICG-FA may not accurately display its perfusion. However, dynamic visualization of the conduit characterizes the perfusion by introducing a target time, which can better identify the optimal perfusion area. Kumagai (15) found that the leakage rate was 0% when the anastomosis was implemented in the area where the ICG perfusion was detected by the FA within 90 s. The rate of AL was a significantly lower than the 33% in the group that anastomosis was performed in areas where FA detected ICG perfusion after 90 s. Furthermore, Noma (23) recently reported that if perfusion was visualized within 20 s, the anastomosis was performed in this area, whereas if anastomotic areas were perfused within 30 s, further mobilization was performed prior to anastomosis implementation. The study found that the AL rates in patients who received the ICG protocol were significantly lower than in those assessed before protocol initiation (8.8% vs. 22%, p = 0.03). In our study, we chose 60 s after injection as a target time to identify the optimal zone for perfusion; this was because the study showed that 30% of the conduits were perfused in less than 60 s and this time was found to be clinically feasible (15). Second, in our previous clinical trials, we found that approximately 20 s after injection, blood flow was confirmed by ICG-FA, and vascular perfusion was well visualized within 60 s. After 60 s, the fluorescence imaging of the gastric wall was jagged and uneven, which was not suitable to identify anastomosis area. Therefore, we hypothesized that the risk of AL might be minimized if anastomosis was created in the area that was enhanced within 60 s; subsequently, we found that anastomosis in an area of the conduit where ICG perfusion was detected by FA within 60 s resulted in a lower leak rate.

There are some limitations to this study. First, this was a retrospective cohort study in a single institution, and the number of patients was limited in each group. To address this, we are now prospectively enrolling patients for randomized controlled assessment. Second, we did not evaluate blood perfusion of the conduit after anastomosis and did not understand the effect of anastomosis surgery on the regional blood supply. Third, ICG assessment was dependent on vascular perfusion via ICG-FA within 60 s; we need to establish an objective criterion for the speed of the ICG fluorescence stream in the gastric conduit. Lastly, ICG-FA requires highly specialized equipment that can be expensive and difficult to acquire.

In conclusion, for the assessment of esophageal conduits, ICG-FA has the potential to reduce the rate of AL in McKeown MIE. Further prospective studies are needed to confirm the usefulness of intraoperative ICG-FA for reducing the rate of AL.

## Data Availability Statement

The original contributions presented in the study are included in the article/**Supplementary Material**. Further inquiries can be directed to the corresponding author.

## Ethics Statement

The studies involving human participants were reviewed and approved by Research Ethics Committee of Sir Run Run Shaw Hospital, School of Medicine, Zhejiang University. The patients/participants provided their written informed consent to participate in this study. Written informed consent was obtained from the individual(s) for the publication of any potentially identifiable images or data included in this article.

## Author Contributions

Collection of data: R-JL, Z-YZ, Z-FH, YX, Y-ZW, PC. Analysis of data: R-JL, PC. Writing of this paper: R-JL. General supervision of the research group: R-JL, Z-YZ. All authors contributed to the article and approved the submitted version.

## Conflict of Interest

The authors declare that the research was conducted in the absence of any commercial or financial relationships that could be construed as a potential conflict of interest.
